# Big Brain Data Initiative in Universiti Sains Malaysia: Challenges in Brain Mapping for Malaysia

**DOI:** 10.21315/mjms2020.27.4.1

**Published:** 2020-08-19

**Authors:** Sharifah Aida Shekh Ibrahim, Nurfaten Hamzah, Athirah Raihanah Abdul Wahab, Jafri Malin Abdullah, Nurul Hashimah Ahamed Hassain Malim, Putra Sumari, Zamzuri Idris, Ariffin Marzuki Mokhtar, Ab Rahman Izaini Ghani, Sanihah Abdul Halim, Salmi Ab Razak

**Affiliations:** 1 Department of Neurosciences, School of Medical Sciences, Universiti Sains Malaysia, Kubang Kerian, Kelantan, Malaysia; 2 Department of Neurosciences, Hospital Universiti Sains Malaysia, Universiti Sains Malaysia, Kubang Kerian, Kelantan, Malaysia; 3 Brain and Behaviour Cluster, School of Medical Sciences, Universiti Sains Malaysia, Kubang Kerian, Kelantan, Malaysia; 4 School of Computer Sciences, Universiti Sains Malaysia, Pulau Pinang, Malaysia; 5 Department of Anesthesiology, School of Medical Sciences, Universiti Sains Malaysia, Kubang Kerian, Kelantan, Malaysia; 6 Department of Anesthesiology, Hospital Universiti Sains Malaysia, Universiti Sains Malaysia, Kubang Kerian, Kelantan, Malaysia; 7 Unit of Neurology, Department of Medicine, School of Medical Sciences, Universiti Sains Malaysia, Kubang Kerian, Kelantan, Malaysia; 8 Unit of Paediatric Neurology, Department of Paediatrics, School of Medical Sciences, Universiti Sains Malaysia, Kubang Kerian, Kelantan, Malaysia

**Keywords:** Big Data, Big Brain Data, brain mapping, neurosciences, initiative, neuroimaging

## Abstract

Universiti Sains Malaysia has started the Big Brain Data Initiative project since the last two years as brain mapping techniques have proven to be important in understanding the molecular, cellular and functional mechanisms of the brain. This Big Brain Data Initiative can be a platform for neurophysicians and neurosurgeons, psychiatrists, psychologists, cognitive neuroscientists, neurotechnologists and other researchers to improve brain mapping techniques. Data collection from a cohort of multiracial population in Malaysia is important for present and future research and finding cure for neurological and mental illness. Malaysia is one of the participant of the Global Brain Consortium (GBC) supported by the World Health Organization. This project is a part of its contribution via the third GBC goal which is influencing the policy process within and between high-income countries and low- and middle-income countries, such as pathways for fair data-sharing of multi-modal imaging data, starting with electroencephalographic data.

## Introduction

The Fourth Industrial Revolution loads our life with tons of data (1). These data that require routine collection, storage, processing and analysis (2). The support of technological advances, it has led to the concept of ‘Big Data’ that describe data that is large and massive (1).

### An Overview of Big Data Definition

The needs of Big Data analytics is steadily increasing in various field as it can be used in remodel operational processes of an organisation. The term of Big Data refers to a collection of large volume of both structured and unstructured data for the purpose of identifying unknown relationships or other informative attributes by using computational analysis that often involving advanced machine learning algorithms (3). It is an information assets that is often characterised by three properties which are high volume, velocity and variety. It requires specific technologies and analytical methods for its transformation into meaningful values (4). The challenges when dealing with Big Data include capture, curation, storage, search, sharing, transfer, analysis and visualisation (5). Although Big Data does not associate to any specific volume of data, Big Data storage often involve terabytes, petabytes and even exabytes of data captured over time (6).

In health research, there is a specific definition of Big Data proposed by the Health Directorate of the Directorate-General for Research and Innovation of the European Commission. The definition Big Data in health encompasses high volume, high diversity biological, clinical, environmental and lifestyle information collected from single individuals to large cohorts, in relation to their health and wellness status, at one or several time points (2).

### The Importance of Big Data in Brain, Mind and the Neurosciences

Information has been the key to build better organisations and new developments. The organisation can optimally organise themselves to deliver the best outcomes if they have more information. That is why data collection is a significant part for every organisation (1). In healthcare, Big Data can be used in the prediction of diseases outcome, prevention of co-morbidities, mortality, premature deaths and disease development, improve treatment and quality of life and reduce the cost of medical treatment (7).

Today, patients want to participate in their health decision-making about their healthcare options or choices. This Big Data will help patients with providing up-to-date information to assist them to make the best decision and to comply with the medical treatment.

In brain, mind and neurosciences, Big Brain Data can lead to important discoveries; particularly, it can help us to understand the brain’s structure and function, identify new biomarkers of brain pathology and increase the performance of neurotechnological devices (such as brain-computer interfaces [BCIs]) (3). This growing Big Data would also be of much use for the advanced machine learning algorithms, specifically artificial neural networks for deep learning and related methods.

## Big Brain Data Initiative in Universiti Sains Malaysia with the Global Brain Consortium

Big Brain Data refers to the recording, collection and analysis of an individual brain data on a large scale by using neurotechnologies, like electroencephalogram (EEG), magnetoencephalography (MEG), near-infrared spectroscopy, magnetic resonance imaging (MRI) and functional magnetic resonance imaging (fMRI) (3).

The Big Data project on retrospective and prospective data for data hospital (pilot neuro) in Universiti Sains Malaysia (USM) was started on the 14th August 2017 (8). It was led by the Brain and Behaviour Cluster, School of Medical Sciences (previously known as Centre for Neuroscience Service and Research) in collaboration with the School of Computer Sciences with three objectives. The main objective was to establish a centralised clinical data storage platform for future references and research. The other two objectives are to create digital (paperless) access and data availability that can be accessed at university level by students, lecturers, clinicians and researchers.

**Figure 1:**
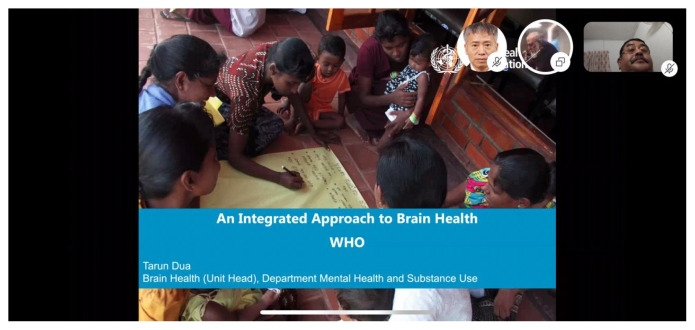
Professor Dato’ Dr Jafri Malin Abdullah joined the GBC Workshop 2020—online web conferencing. The topic ‘An Integrated Approach to Brain Health’ was presented by Dr Tarun Dua, Brain Health (Unit Head), Department Mental Health and Substance Use, WHO

**Figure 2:**
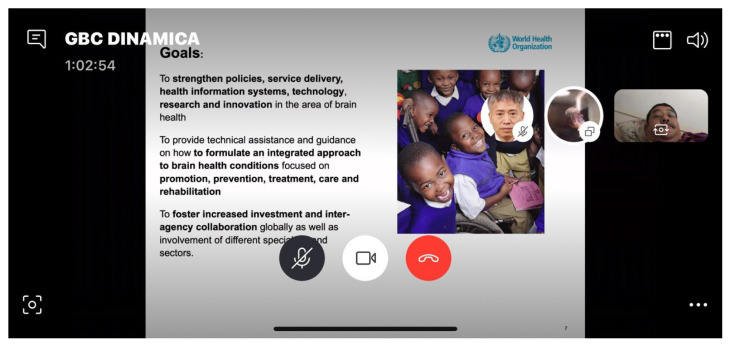
Dr Tarun Dua presented slides on the three goals of an Integrated Approach to Brain Health

**Figure 3:**
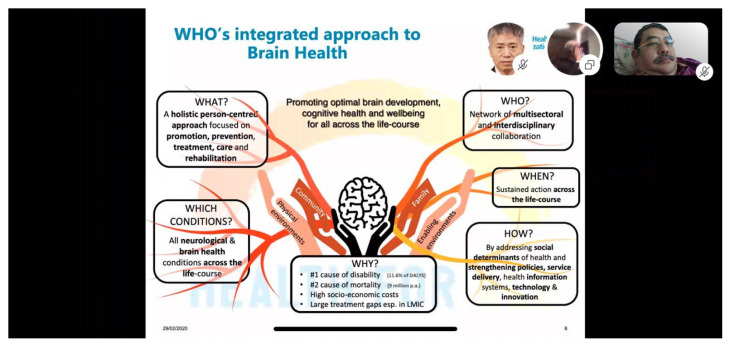
Promoting optimal brain development, cognitive health and well-being for all across the life-course

**Figure 4:**
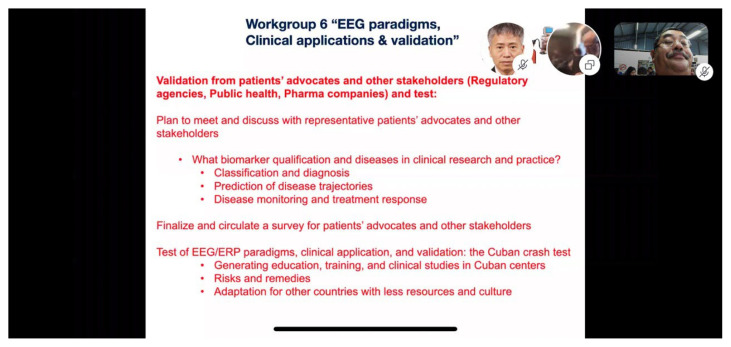
Workgroup 6 of the GBC 2020 presented validation from patients’ advocates and other stakeholders (regulatory agencies, public health, pharma companies) and test from EEG paradigms, clinical applications and validation topic

**Figure 5:**
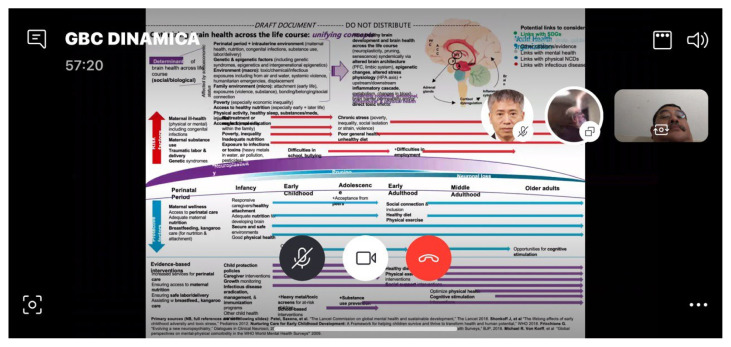
Optimising brain health across the life course: unifying concepts in terms of determinat of brain health across the course (social/biological), risk factors and protective factors

This project was developed to contribute to the third Global Brain Consortium (GBC) goals which was influencing the policy process within and between high-income countries and low-and middle-income countries, such as pathways for fair data-sharing of multi-modal imaging data, starting with EEG (9). GBC is a global collaboration of brain, mind and neuroscientists across borders and disciplines to build a fluid and connected global research community that is better able to advance equitable solutions to priority health challenges worldwide (10). Malaysia was honoured to be invited to attend the GBC Plenary Meeting 2019 that was held on 9th May to 10th May 2019 at Montreal Neurological Institute-Hospital, Canada supported World Health Organization (WHO). Malaysia was represented by Professor Dato’ Dr Jafri Malin Abdullah, the Chairman of Brain and Behaviour Cluster, School of Medical Sciences, USM. Continuation from that, he attended the online GBC Workshop 2020 from 27th February to 28th February 2020 at Varadero, Cuba in order to improve the international standards of the USM Big Data Project as shown in Figures 1 to 5.

The retrospective of MEGs, EEGs and MRIs were collected from year 2001 to 2018 from Hospital USM by two researchers, Sharifah Aida Shekh Ibrahim and Nurfaten Hamzah, from Health Campus, USM, meanwhile the interface and the storage platform was developed and design by Associate Professor Dr Nurul Hashimah Ahamed Hassain Malim, a lecturer from the School of Computer Sciences, USM. Currently, the project is on the testing phase.

**Figure 6:**
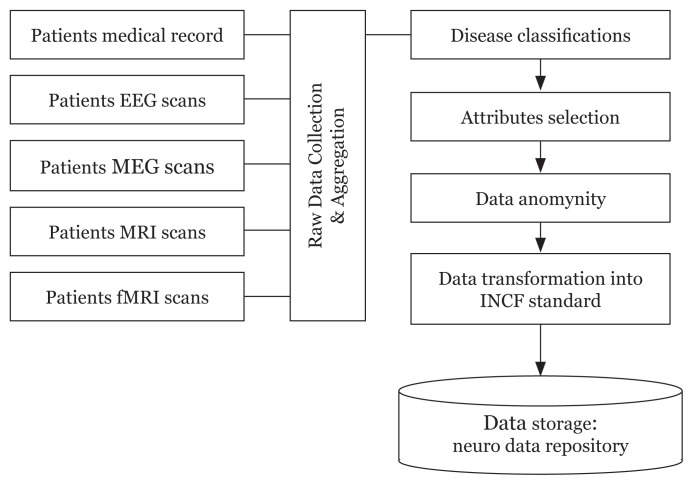
The data pre-processing (curation) pipeline © USM (8) starts with collection of records (medical records and scanning records) and followed by collection and aggregation, disease classifications, attribute selection, data anonymity, data transformation into international neuroinformatics coordinating facility standard and neuro data repository

Figure 6 shows the data pre-processing (curation) pipeline that had been established to understand the formats of data and the degree of aggregation needed from the various sources of input. Meanwhile, Table 1 shows the latest progress on Big Brain Data Initiative of USM.

## Benefits of Big Brain Data to Researchers/Clinicians in Malaysia and the World

There are several benefits to researchers/clinicians of Big Brain Data such as for our better understanding of the brain’s anatomy including structure and it’s function, identifying new biomarkers of brain pathology, neurotechnological devices performance improvement for example BCIs (3) and opportunity to explore and implementing of ‘internet of things’ (IOT) in health industries (1).

## An Overview of Brain Mapping

Brain mapping techniques have proven to be vital in understanding the molecular, cellular and functional mechanisms of the brain. Normal anatomical imaging can provide structural information on certain abnormalities in the brain. However there are many neurological disorders for which only structure studies are not sufficient. In such cases it is required to investigate the functional organisation of the brain. Brain mapping techniques can help in deriving useful and important information on these issues (11).

According to the Society for Brain Mapping and Therapeutics (SBMT), United States of America, brain mapping is clearly defined as the study of the structural and functional of the brain and spinal cord through the use of imaging. Mapping brain structural and functional connections through the whole brain is important for understanding brain mechanisms and the physiological bases of brain diseases (12). The development of high-resolution neuroimaging and multielectrode electrophysiological recording can be considered as a part of brain mapping that can help brain, mind and neuroscientists to map what the brain looks like as disease progress or as treatments work. The commonly neurotechnologies used are positron emission tomography and fMRI along with EEG, electrocorticography, MEG and, most recently, optical imaging with near-infrared spectroscopy (13).

## The Challenges in Brain Mapping

Four common challenges in brain mapping has been identified which are scale, complexity, speed and integration.

- Scale – large numbers of neurons and synapses, human brain simulation would push exascale computers to work beyond limit.
- Complexity – almost limitless set of parameters are require to produce a biologically faithful simulation of the brain. Brain’s extracellular interactions and molecular-scale processes such as receptor binding are examples of details which not incorporated into simulation models.
- Speed – currently there is no technology can support large-scale simulations faster than in real time, normally models run slower. It is because human’s brain processes such as development and learning took years or decades to perform.
- Integration – combination of smaller models of brain regions are needed in order to model functions that involve brain-wide networks. Top-down and bottom-up models must be integrated, for example, those that cast the brain as a hypothesis testing system and biophysical model that represent simulations (14–19).

## Conclusion

The Big Brain Data Initiative in USM with objectives to establish a centralised clinical data storage platform for future references and research, creating digital (paperless) access and data availability which can be access at university level by students, lecturers, clinicians and researchers is reaching to the testing phase.

Moving to the next stage is to implement the brain mapping techniques for understanding the molecular, cellular and functional mechanism of the brain. Four commons challenges will be faced by researchers are scale, complexity, speed and integration. The brain-techniques and computer technologies should play their roles simultaneously in order to overcome the challenges.

## Figures and Tables

**Table 1:** Raw data collection and aggregation of EEG and MEG are 100% collected. While MRI data collection is 15% because the number and size of the data is huge. The fMRI data collection is ongoing

	Raw data collection and aggregation (%)	Record date range	Disease classification (%)	Attributes selection (%)	Data anonymity (%)	Data transformation (%)	Number of records
EEG	100	2001–March 2018	50	80	100	2	4214
MEG	100	2008–February 2018	60	90	100	30	190
MRI	15	2002–October 2018	50	60	10	1	8876
fMRI	0	NA	0	0	0	0	19
